# Reagent-Less and Robust Biosensor for Direct Determination of Lactate in Food Samples

**DOI:** 10.3390/s17010144

**Published:** 2017-01-13

**Authors:** Iria Bravo, Mónica Revenga-Parra, Félix Pariente, Encarnación Lorenzo

**Affiliations:** 1 Departamento de Química Analítica y Análisis Instrumental, Universidad Autónoma de Madrid, Cantoblanco, 28049 Madrid, Spain; iria.bravo@uam.es (I.B.); monica.revenga@uam.es (M.R.-P.); felix.pariente@uam.es (F.P.); 2 Instituto Madrileño de Estudios Avanzados en Nanociencia (IMDEA-Nanociencia), Faraday, 9, Campus UAM, Cantoblanco, 28049 Madrid, Spain; 3 Institute for Advanced Research in Chemical Sciences (IAdChem), Universidad Autónoma de Madrid, 28049 Madrid, Spain

**Keywords:** Schiff base tetradentate ligand modified gold nanoparticles, lactate determination, enzymatic biosensor, gold nanoparticles, lactate oxidase biosensor

## Abstract

Lactic acid is a relevant analyte in the food industry, since it affects the flavor, freshness, and storage quality of several products, such as milk and dairy products, juices, or wines. It is the product of lactose or malo-lactic fermentation. In this work, we developed a lactate biosensor based on the immobilization of lactate oxidase (LOx) onto *N*,*N*′-Bis(3,4-dihydroxybenzylidene) -1,2-diaminobenzene Schiff base tetradentate ligand-modified gold nanoparticles (3,4DHS–AuNPs) deposited onto screen-printed carbon electrodes, which exhibit a potent electrocatalytic effect towards hydrogen peroxide oxidation/reduction. 3,4DHS–AuNPs were synthesized within a unique reaction step, in which 3,4DHS acts as reducing/capping/modifier agent for the generation of stable colloidal suspensions of Schiff base ligand–AuNPs assemblies of controlled size. The ligand—in addition to its reduction action—provides a robust coating to gold nanoparticles and a catalytic function. Lactate oxidase (LOx) catalyzes the conversion of l-lactate to pyruvate in the presence of oxygen, producing hydrogen peroxide, which is catalytically oxidized at 3,4DHS–AuNPs modified screen-printed carbon electrodes at +0.2 V. The measured electrocatalytic current is directly proportional to the concentration of peroxide, which is related to the amount of lactate present in the sample. The developed biosensor shows a detection limit of 2.6 μM lactate and a sensitivity of 5.1 ± 0.1 μA·mM^−1^. The utility of the device has been demonstrated by the determination of the lactate content in different matrixes (white wine, beer, and yogurt). The obtained results compare well to those obtained using a standard enzymatic-spectrophotometric assay kit.

## 1. Introduction

l-lactate is a metabolite generated during anaerobic glucose metabolism. Its production involves an increase in the proton concentration inside the cells. l-lactate levels in blood range from 0.5 to 1.5 mM in normal people at rest, and can increase to 12.0 mM during exercise [1]. Under extreme conditions of excessive exercise, lactate levels can become as high as 25 mM. In this case, the cellular proton buffering may be exceeded. Hence, the cell pH decreases, and this may result in cell lactic acidosis, which disrupts the performance of the muscles [2]. Therefore, knowledge of personal lactic threshold is very important to perform any physical activity, and this is the reason why sport medicine requires the monitoring of l-lactic levels in order to evaluate the so-called lactic threshold [3], which indicates the physical training level of an athlete. Lactate levels in plasma depend on the balance between factors that produce lactate and lactate clearance factors [4]. Any imbalance between the production and clearance of lactate may lead to the lactic acidosis. The increase of lactate levels in blood may be due to the presence of very diverse pathologies, such as haemorrhagic shock or pulmonary embolism [4], cardiogenic [5] or endotoxic shocks [6], respiratory failure [7], liver diseases [8], and others. High levels of lactate are also the main cause of acidification in the microenvironment of cancer cells and tumors [9], due to the imbalance between the dynamics of the production and consumption of lactate, characteristic for these types of cells and tissues [10]. Thus, the importance of determining lactate level in medical care is evident because the blood lactate level is a valuable indicator of both fitness and clinical states.

Lactic acid fermentation is an important process in the food industry. Lactate levels may indicate the stability, freshness, and quality of many fermented dairy products such as milk, yogurt, and creams, as well as raw meat, fruits, and vegetables [11]. In wine production, the malolactic fermentation carried out by bacteria present in the grapes and the must is very important. This fermentation takes place after the alcoholic fermentation, and converts the l-malic acid to l-lactic reducing the wine acidity, and leading to an improvement of the taste and flavor of the final product. The control of this secondary fermentation is very important in the quality of wines, and requires a continuous monitoring of the levels of lactic and malic acids present in the young wine [11].

The examples described above show the great interest that exists in monitoring lactate concentration for clinical diagnosis, intensive care, in the food industry, and in food control, among others. To achieve these goals, there is a great demand for devices capable of determining lactate in a simple and direct way with rapid response, high specificity, low cost, and minimal or no sample preparation. In this regard, a variety of methods have been described, using techniques such as amperometry [12], potentiometry [13], high-performance liquid chromatography [14], fluorometry [15], chemiluminescence [16], microwave sensing [17], holography [18], and magnetic resonance spectroscopy [19]. Because of their simplicity, portability, and easy integration in different devices, amperometric biosensors have reached the most significant development—in particular when reagent-less biosensors based on disposable electrodes are employed.

**Scheme 1:**
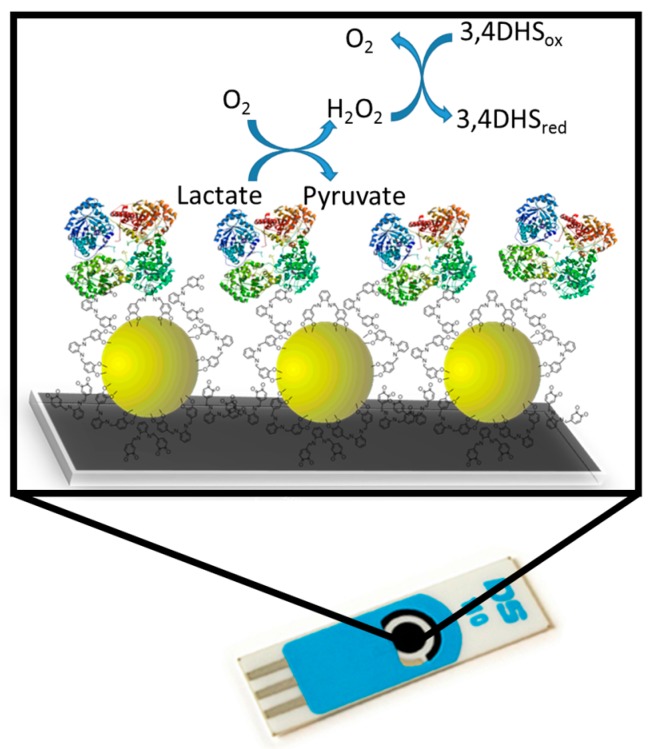
Scheme of the biosensing platform. 3,4DHS: *N*,*N*′-Bis(3,4-dihydroxybenzylidene)-1,2-diaminobenzene.

Different biosensors for lactate monitoring have been reported based on immobilized lactate oxidase [20,21,22,23]. Bienzyme systems such as lactate oxidase/lactate dehydrogenase (LOx/LDH) have also been described [24]. In these configurations, the enzyme was immobilized in a polymeric matrix inside the electrode material. Recently, in order to improve the biosensor response, lactate biosensors using nanoparticles have been described [25,26,27,28,29]. In general, these devices showed linearity in the range of μM to mM lactate concentration.

l-lactate oxidase and lactate dehydrogenase have been the enzymes commonly used in the design of l-lactate biosensors due to the favorable kinetics of the enzymatic reactions, the stability of the immobilized enzymes, and the simple design of the biosensors. In fact, LOx is usually preferred over LDH because its reaction involves O_2_ and H_2_O_2_ that can be amperometrically detected. However, the oxidation of H_2_O_2_ requires a high overpotential. A strategy employed to overcome this problem is the addition of redox mediators to the solution, and in order to simplify the systems, an improved strategy is to include the mediator in the device development.

l-lactate oxidase is a globular flavoprotein with a mean diameter of 5 nm that can be obtained with an acceptable degree of purity from various microorganisms, such as *Pediococcus* and *Aerococcus viridians* [30]. In the presence of molecular oxygen, the enzyme catalyzes the oxidation of l-lactate to pyruvate, yielding hydrogen peroxide, which is electrochemically active and can be oxidized or reduced on the electrode surface giving rise to a current which is proportional to the concentration of lactate present in the sample [31,32]. As can be seen in Scheme 1, the electrons involved in the reaction are transferred from the substrate (l-lactate) to the oxidized form of the enzyme cofactor (Flavin Adenine Dinucleotide, FAD) present in the enzyme structure. To regenerate the enzyme catalytic site, molecular oxygen takes the electrons from the reduced cofactor, turning into hydrogen peroxide. The flow of electrons from the substrate to the electrode surface can be enhanced by the presence of highly conductive nanomaterials in the sensing interface, which can act as tiny conductive centers, allowing kinetic barriers to be eliminated, and yielding more sensitive electrochemical responses [33].

Recently, we reported the use of a multi-tasking *N*,*N*′-Bis(3,4-dihydroxybenzylidene) -1,2-diaminobenzene Schiff base tetradentate ligand (3,4DHS) as reductant, stabilizer, and catalyst in a new concept of gold nanoparticles (AuNPs) synthesis [34]. This ligand contains the quinone/hydroquinone functional group and is capable of reducing HAuCl_4_ in water, also acting as a capping agent for the generation of stable colloidal suspensions of Schiff base ligand–AuNPs assemblies of controlled size by providing a robust coating to AuNPs within a unique reaction step. These 3,4DHS–AuNPs assemblies—deposited on carbon electrodes—show a potent electrocatalytic effect towards hydrazine oxidation and hydrogen peroxide oxidation/reduction [34]. Following these previous studies, the present work takes the preparation of 3,4DHS–AuNPs assemblies one-step further by using them for the construction of a new l-lactate biosensor. This is based on the co-immobilization of l-lactate oxidase along with 3,4DHS–AuNPs onto a screen-printed carbon electrode giving rise to a reagent-less biosensor, in an effort to simplify the development of point-of-care lactate analysis systems.

The biosensor response was optimized in terms of enzyme loading, solution pH, and the effect of potentially interfering substances. Finally, the developed biosensor has been applied to the determination of lactate in food and beverage samples. The results were validated by comparing with those obtained with a commercial enzymatic kit.

## 2. Materials and Methods

### 2.1. Reagents and Apparatus

Hydrogen tetrachloroaurate (III) trihydrate (HAuCl_4_·3H_2_O, ≥99.9% trace metals basis) and l-(+)-lactic acid lithium salt 97% were obtained from Aldrich Chemical Co. (Milwaukee, WI, USA). Lactate oxidase (LOx, EC 232-841-6 from *Pediococcus* species) lyophilized powder was obtained from Sigma Chemical Co. (St. Louis, MO, USA). Stock solutions were prepared 200 U·mL^−1^ in 0.1 M phosphate buffer (pH 7.0) and stored at −30 °C. Under these conditions, the enzymatic activities remain stable for several weeks. The enzymatic assay kit for lactate determination (K-LATE 07/14) was purchased from Megazyme (Ireland). Other chemicals used in this work were reagent grade quality and used as received without further purification. MilliQ water (Millipore^®^, Darmstadt, Germany) was used throughout.

*N*,*N*′-Bis(3,4-dihydroxybenzylidene)-1,2-diaminobenzene (3,4DHS) was prepared as previously reported [35] by condensation of 1,2-phenylendiamine with the 3,4-dihydroxybenzaldehyde isomer in a 1:2 molar stoichiometric ratio. The resulting compound was recrystallized from hot methanol. Stock solutions of 3,4DHS (typically 2.0 mM) were prepared just prior to use by dissolving the solid in dimethylformamide.

All electrochemical measurements were carried out using an Autolab potentiostat/galvanostat type PGSTAT 302N (Eco Chemie, Utrecht, The Netherlands) using the software package GPES 4.9. Integrated screen-printed carbon electrodes (4 mm diameter, SPCEs) from DropSens were used. They include a silver pseudoreference electrode and a carbon counter electrode. A 1.5 mL home-made glass electrochemical cell was employed to carry out the measurements. All experiments were carried out in 0.1 M phosphate buffer (pH 7.0) at 25 °C.

### 2.2. Procedures

#### 2.2.1. Synthesis of 3,4DHS Capped Gold Nanoparticles (3,4DHS–AuNPs)

3,4DHS–AuNPs were synthesized as previously reported [34] by mixing aqueous HAuCl_4_ with 3,4DHS solution, and chemical reduction was left to proceed for 24 h at room temperature. The 3,4DHS capped AuNPs thus formed were separated by centrifugation at 9000 rpm (7700 g) and washed with water to remove excess 3,4DHS.

The size and concentration of 3,4DHS–AuNPs were estimated from the ratio of absorbance measured at the surface plasmon band (~520 nm) and the absorbance at 450 nm, as described by Haiss et al. [36]. The particle size value was confirmed by transmission electron microscopy [34]. Thus, a stock solution of 17.7 nM (33 ± 3 nm) 3,4DHS–AuNPs was employed.

#### 2.2.2. Biosensor Preparation

Screen-printed carbon electrodes (SPCEs) were modified by transferring of 4 μL of the 3,4DHS–AuNPs solution onto the working electrode surface (3,4DHS–AuNP/SPCE) followed by air-drying at room temperature. Once dried, 5 μL of LOx (1 U) were deposited on the 3,4DHS–AuNP/SPCE surface and placed for 1 h at 4 °C to assemble the protein on the electrode surface (LOx/3,4DHS–AuNP/SPCE).

#### 2.2.3. Determination of Lactate in Food Samples

Lactate was determined in food samples using the developed amperometric biosensor. Results were compared with those obtained using a commercial enzymatic assay kit following the procedure described by the manufacturer. White wine, beer, and yogurt were purchased in a local store and were analyzed by standard addition method without any pre-treatment other than dilution in 0.1 M phosphate buffer (pH 7.0).

## 3. Results and Discussion

### 3.1. Lactate Oxidase Biosensor Development

The modification of AuNPs with electroactive substances can be of great interest in designing new nanostructured platforms with applications in catalytic processes, and in the construction of biosensors [37]. In a previous work, we reported the multi-tasking *N*,*N*′-Bis(3,4-dihydroxybenzylidene)-1,2-diaminobenzene Schiff base tetradentate ligand (3,4DHS) as reductant, stabilizer, and catalyst in a new concept of AuNPs synthesis. Once these assemblies were deposited on screen-printed carbon electrodes, the resulting nanostructured platforms exhibited excellent electrocatalytic activity towards the oxidation and the reduction of peroxide in alkaline medium [34]. In this work, we have gone a step further and have used these platforms to build a portable lactate biosensor based on lactate oxidase in which the lactate is oxidized to pyruvate and hydrogen peroxide in presence of oxygen. The enzymatically generated H_2_O_2_ is electrocatalytically oxidized at the 3,4DHS–AuNPs modified electrode, giving rise to the analytical signal (Scheme 1).

**Figure 1:**
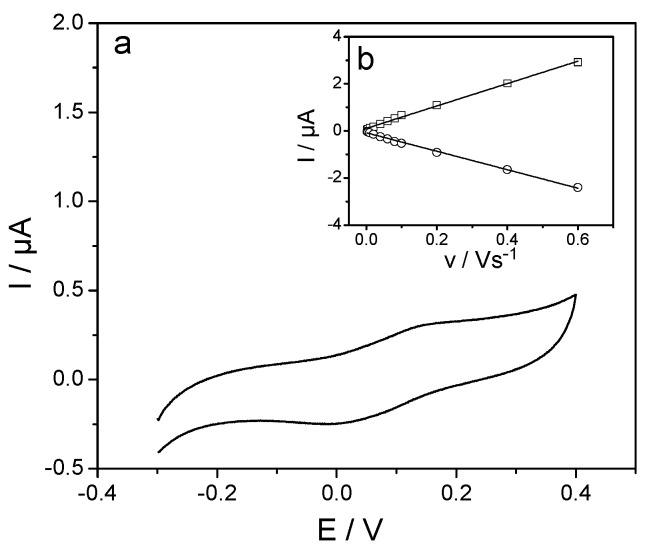
(**a**) Cyclic voltammogram of screen-printed carbon electrode modified with 3,4DHS capped gold nanoparticles (3,4DHS–AuNP/SPCE) in 0.1 M phosphate buffer (pH 7) at 0.04 V·s^−1^; The inset (**b**) shows the dependence of the anodic and cathodic peak current on the scan rate.

**Figure 2:**
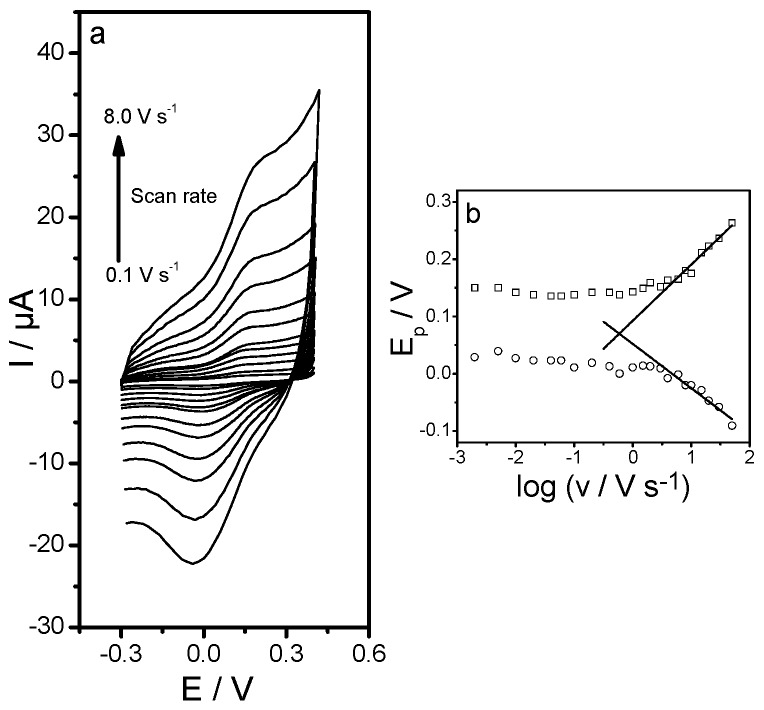
(**a**) Cyclic voltammetric response of 3,4DHS–AuNP/SPCE in 0.1 M phosphate buffer (pH 7) at different scan rates; (**b**) Laviron’s plot showing the dependence of the peak potential on the logarithm of scan rate.

At a first step, the electrochemical behavior of the screen-printed carbon electrode modified with 3,4DHS capped gold nanoparticles (3,4DHS–AuNP/SPCE) was studied using cyclic voltammetry in 0.1 M phosphate buffer (pH 7). As can be seen in Figure 1a, a well-defined redox couple with anodic and cathodic peak potentials centered at +0.13 V and +0.02 V, respectively, was observed. This redox couple is ascribed to the quinone/hydroquinone functional groups present in the 3,4DHS–AuNPs. The formal potential (E = +0.08 V) was practically constant at low scan rates, and the anodic and cathodic peak currents showed a linear dependence with the scan rate in the range studied, as anticipated for a redox couple confined on the electrode surface (Figure 1b). The peak-to-peak separation (ΔEp) was 0.11 V (far from the 0 value anticipated for a reversible redox process confined on the electrode surface), and increased significantly for scan rates higher than 1.0 V·s^−1^, suggesting severe kinetic limitations in the charge transfer (Figure 2a). The anodic and cathodic peak potentials were plotted vs. log (v; scan rate) in a typical Laviron’s plot [38] (Figure 2b). At scan rates higher than 5 V·s^−1^, the peak potential depicts a linear dependence with log (v). From the ratio of the slopes of these straight lines, according to Laviron’s equation Ep = E° + (RT/αnF) [ln(RT·k_s_/αnF) − lnv], the electron-transfer coefficient (α) and the apparent surface electron-transfer rate constant (k_s_) were found to be 0.50 and 47 s^−1^, respectively.

**Figure 3:**
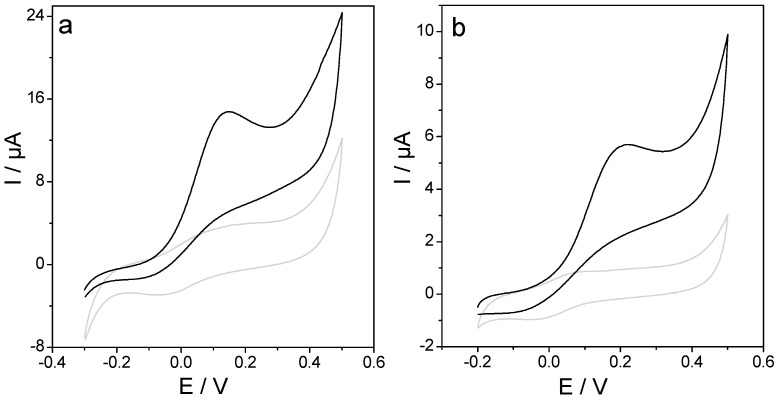
(**a**) Cyclic voltammogram of 3,4DHS–AuNP/SPCE in 0.1 M phosphate buffer (pH 7) in the absence (grey line) or presence (black line) of 1.0 mM of H_2_O_2_; (**b**) Cyclic voltammogram of lactate oxidase (LOx)/3,4DHS–AuNP/SPCE in 0.1 M phosphate buffer (pH 7) in the absence (dotted line) or presence (solid line) of 0.5 mM lactate. Scan rate: 0.01 V·s^−1^.

The electrocatalytic behavior of the 3,4DHS–AuNP/SPC modified electrodes toward the oxidation of H_2_O_2_ at pH 7—the optimal for lactate oxidase enzyme function—was studied. Figure 3a shows the cyclic voltammograms of 3,4DHS–AuNP/SPCE in 0.1 M phosphate buffer (pH 7) in the absence and presence of 1.0 mM of H_2_O_2_ recorded at 0.01 V·s^−1^. In the absence of H_2_O_2_, a well-defined electrochemical response (Figure 3a, grey line) ascribed to the oxidation/reduction of the hydroquinone/quinone moieties of the 3,4DHS was observed. However, in the presence of H_2_O_2_, there was a dramatic increase in the anodic peak current, and practically no current was observed in the reverse (cathodic) scan (Figure 3a, black line). This behavior is consistent with a very strong electrocatalytic effect. Thus, 3,4DHS–AuNP/SPCE has an excellent electroacatalytic activity towards the oxidation of H_2_O_2_, and facilitates electrochemical detection of this compound at lower potential.

The 3,4DHS–AuNP/SPCE platform was used to develop an amperometric lactate biosensor based on the quantification of the H_2_O_2_ liberated during the enzymatic reaction. The biosensor was developed by coupling the lactate oxidase enzyme to the 3,4DHS–AuNP/SPCE platform, as described in the experimental section. The cyclic voltammetric biosensor response (LOx/3,4DHS–AuNP/SPCE) in the presence and absence of the substrate (lactate) was used to assess its catalytic activity (Figure 3b). In the absence of lactate (grey line), the well-behaved redox response of 3,4DHS–AuNP was readily apparent. Upon the addition of lactate (to a final concentration of 0.5 mM), there was an increase in the anodic peak current due to electrocatalytic oxidation of the H_2_O_2_ generated in the enzymatic reaction. In addition, a decrease in the cathodic peak current was observed (black line). To confirm the role of the enzyme in the catalytic response to substrate, 3,4DHS–AuNP/SPC modified electrodes without immobilized LOx were immersed in 0.1 M phosphate buffer solution (pH 7.0). As one would expect, upon addition of lactate, no catalytic waves were observed.

**Figure 4:**
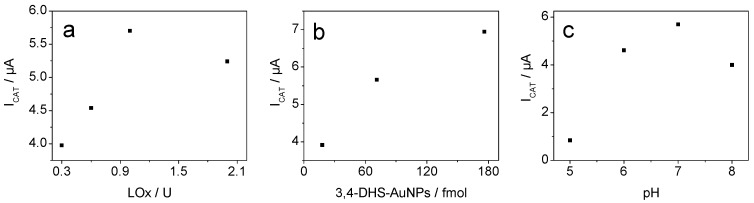
(**a**) Biosensor current response at different loadings of LOx enzyme in 0.1 M phosphate buffer (pH 7) containing 0.5 mM of lactate. Effect of (**b**) 3,4DHS–AuNP concentration and (**c**) pH on the response to 0.5 mM lactate of a biosensor prepared with 1.0 U of LOx.

The amount of both LOx and 3,4DHS–AuNPs in the biosensor development was optimized. For this purpose, different biosensors with increasing amounts of both components were prepared, and their responses to lactate were obtained. The biosensor response increased as the units of enzyme included in the biosensing layer increased from 0.3 U to 1.0 U (Figure 4a). At higher enzyme loading, there was a decrease in the biosensor response. This is probably due to an excess of protein on the biosensing layer that could prevent the charge transfer towards the electrode surface. In accordance with these results, 1.0 U of LOx was chosen as optimal.

As one would expect, the LOx biosensor response showed a significant dependence on the amount of 3,4DHS–AuNPs deposited on the electrode surface. Biosensors prepared using 20, 70, or 180 fmol of 3,4DHS–AuNPs showed different responses. Although the highest catalytic response was obtained when 180 fmol were employed (Figure 4b), the catalytic efficiency—defined as the ratio between the catalytic current and the current measured for the LOx/3,4DHS–AuNP/SPCE in the absence of lactate (I_CAT_/I_MED_)—was higher when 70 fmol 3,4DHS–AuNPs were employed. Therefore, this amount was chosen for further studies.

The effect of the buffer solution pH on the biosensor response was investigated over the range 5.0–8.0. The results showed that the response increased upon increasing the pH, reaching a maximum value at 7.0, and then the response decreased (Figure 4c). Hence, 0.1 M phosphate buffer (pH 7) was selected for the determination of lactate.

**Figure 5:**
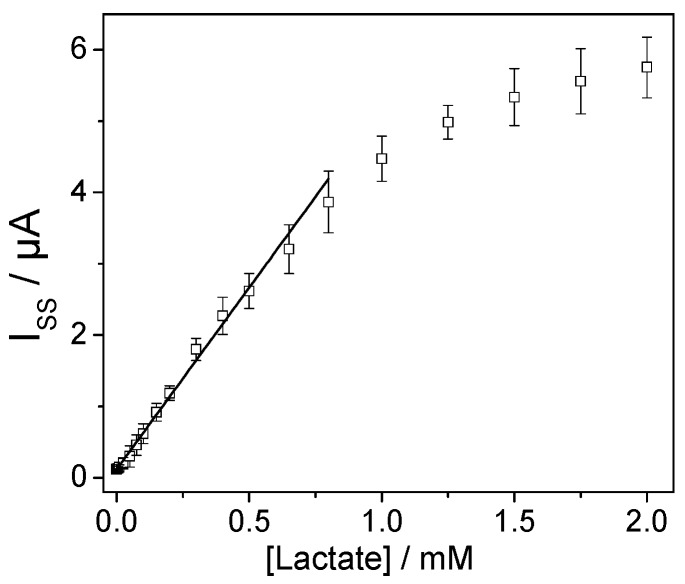
Calibration curve (*n* = 3) obtained from chronoamperometric measurements for LOx/3,4DHS–AuNP/SPCE in 0.1 M phosphate buffer (pH 7) in the presence of increasing amounts of lactate.

Once the biosensor development and work conditions were optimized, its response to lactate was investigated by chronoamperometry, applying a constant potential of +0.3 V. Under the optimal conditions, the biosensor showed a reproducible and stable response to different lactate concentrations. Figure 5 depicts a typical calibration curve, which follows Michaelis–Menten kinetics. This confirms that the biosensor response is controlled by the enzymatic reaction. The analytical properties of the biosensor were obtained from the linear part (up to 800 μM) of the calibration plot. The sensitivity—calculated from the slope of the plot—was found to be 5.1 ± 0.1 μA·mM^−1^. The detection and quantification limits—calculated as the concentration of lactate that gave a signal equal to three and ten times the standard deviation of background current—were found to be 2.6 and 8.6 μM, respectively. The detection limit compares favorably with other previously described nanostructured lactate biosensors based on modified screen-printed electrodes [28,29]. The reproducibility was evaluated comparing the analytical signals obtained using three different biosensors prepared in the same manner. A value of less than 9% was obtained. Finally, the stability was examined by measuring the response of three different biosensors towards 0.5 mM lactate for one month. After this period, the biosensor retained 85% of its original response.

### 3.2. Study of Common Interfering Substances on the Response of Lactate

One of the most important aspects to consider for any analytical application of biosensors is the study of the effect of potential interfering substances present in real samples. Therefore, to test the utility of the proposed biosensor in the determination of the lactate present in different real samples (such as white wine, beer and yogurt), a study of the influence of the most usual interfering substances that may be present in these samples was carried out. For this purpose, the biosensor response was obtained under the optimized experimental conditions in the absence and presence of different concentrations of tartaric acid, citric acid, ascorbic acid, acetic acid, glucose, fructose, methanol, and ethanol (Table 1). The presence of the potential interfering compounds—when they were at the same concentration as the analyte—did not affect the response, except in the case of ascorbic acid, where an increase of about 76% of the signal was observed. However, at lower concentrations, the presence of this compound did not show any effect. Therefore, these results suggest that the proposed device can be used to measure lactate concentrations in the presence of a variety of possible interfering substances. Moreover, other important analytical parameters such as sensitivity and stability compare favorably to other previously described lactate biosensors prepared in a similar manner [28,29,39,40]. However, in this work, a conjugate nanomaterial consisting of AuNPs with a reagent that has an electrocatalytic activity towards the oxidation of H_2_O_2_ has been obtained in a unique process for the first time. This conjugate (3,4DHS–AuNP) can be easily immobilized on a screen-printed electrode and combined with the enzyme LOx to prepare a biosensor that could determine lactate in real samples directly without previous treatment and without the need to add reagents to the sample. This is an advantage compared to other lactate biosensors prepared using different nanomaterials or electrocatalysts.

### 3.3. Determination of Lactate in Real Samples

Finally, the developed biosensor was applied to the determination of lactate in real food samples—in particular, wine, beer, and yogurt. The importance of lactate in dairy products is well known. Moreover, this analyte plays an important role in the quality of wines, since the amount of lactic, malic, citric or succinic acid in wines is important for the provision of a mild and pleasant acidity.

Samples (25 μL of wine, 40 μL of beer, or 10 μL of yogurt) were diluted in 10 mL of 0.1 M phosphate buffer (pH 7.0), and the standard addition method was used in order to minimize matrix effects. The results obtained were compared to those obtained by a commercial enzymatic assay kit based on l-lactate dehydrogenase/glutamate–pyruvate transaminase and photometric measurement at 340 nm of NADH formed during the enzymatic reaction. The results are summarized in Table 2. The average lactate concentration value obtained for three measurements using different biosensors agrees well with that obtained by the commercial enzymatic kit, with the advantage that the experimental procedure is more rapid, direct, and economical when the biosensor is used.

## 4. Conclusions

Screen-printed carbon electrodes nanostructured with 3,4DHS–AuNPs assemblies are excellent electrocatalytic platforms for the oxidation of H_2_O_2_. Once these platforms are coupled to lactate oxidase, they result in reagent-less disposable biosensors for direct determination of lactate in different samples (wine, beer, and yogurt) without the need for tedious and time-consuming pretreatment steps, and with excellent sensitivity and selectivity. The resulting biosensors are stable, and retain their activity for more than one month.

## Figures and Tables

**Table 1:** Interference tests on various compounds for the assay of lactate.

Compound	Current Ratio ^1^	
	1:1	1:0.1
Tartaric acid	1.05	0.97
Citric acid	1.08	1.00
Acetic acid	1.16	0.88
Ascorbic acid	1.76	1.08
Glucose	1.07	0.96
Fructose	1.01	0.99
Methanol	1.12	0.97
Ethanol	1.10	0.94

^1^ Current ratio = I_L+I_/I_L_. I_L+I_: response of 0.50 mM lactate in the presence of interfering compound; I_L_: response of 0.50 mM lactate.

**Table 2:** Determination of lactate in different samples with the biosensor and with a commercial enzymatic assay kit.

	Lactate Content (g/L) ± SD ^1^	Lactate Content (g/L) ± SD ^1^
Sample	(LOx Biosensor)	(Commercial Assay Kit)
White wine	1.7 ± 0.1	1.73 ± 0.02
Beer	0.10 ± 0.02	0.100 ± 0.001
Yogurt	5.4 ± 0.6	5.7 ± 0.4

^1^ SD: Standard deviation of three determinations.
